# Percutaneous Ventricular Assist Devices: New *Deus Ex Machina*?

**DOI:** 10.1155/2011/604397

**Published:** 2011-07-31

**Authors:** Diego Arroyo, Stéphane Cook

**Affiliations:** Cardiology Unit, University Medical Center, University of Fribourg, 1708 Fribourg, Switzerland

## Abstract

The development of ventricular assist devices has broadened the means with which one can treat acute heart failure. Percutaneous ventricular assist devices (pVAD) have risen from recent technological advances. They are smaller, easier, and faster to implant, all important qualities in the setting of acute heart failure. The present paper briefly describes the functioning and assets of the most common devices used today. It gives an overview of the current evidence and indications for left ventricular assist device use in cardiogenic shock and high-risk percutaneous coronary intervention. Finally, extracorporeal life support devices are dealt with in the setting of hemodynamic support.

## 1. Introduction

Severe heart failure whether acute or chronic is a strenuous clinical challenge. Noninvasive management through inotropic support allows frequent clinical improvement, yet one is repeatedly confronted with refractory cases necessitating more invasive support.

The idea of a mechanical assistance first appeared in the 1950s, yet the first device which is the intra-aortic balloon pump (IABP) only appeared in the late 1960s. It remains, today, the most common, cheapest, and easily available cardiac mechanical device. The most frequent use of IABP is cardiogenic shock with data accounting for 20% of all insertions [1]. It is effective in the stabilization of patients, but it does not provide full cardiac support, and improvement of outcome has not been demonstrated [2]. Hemodynamically, it achieves a maximum of increase of cardiac output of 0.5 L/min. Moreover, its reliance is dependant by the intrinsic cardiac function as well as stable rhythm. In light of these facts, growing interest and expertise have been invested in the development of devices thought to supplement the failing heart. Today, a large pallet of ventricular assist devices is used for a wide range of indications; from long-term replacement of failing hearts to bridge-to-transplantation but also, and foremost, in the temporary support of cardiogenic shock (*bridge-to-recovery*) and its prophylactic use in certain invasive coronary or valvular procedures.

**Figure 1:**
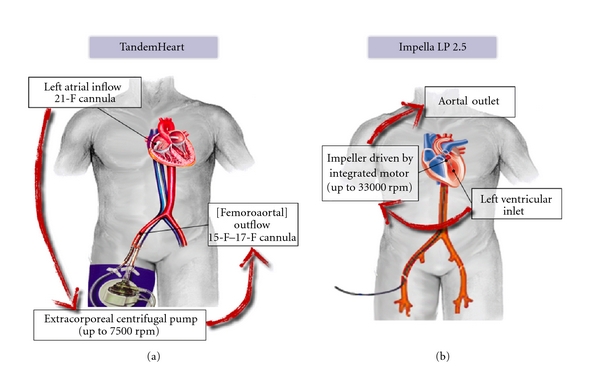
Schematic representation of two commercially available percutaneous ventricular assist devices (VAD). (a) The TandemHeart pVAD consists of a 21 F left atrial inflow cannula, an extracorporeal centrifugal pump rotating at up to 7500 rpm, a femoral outflow cannula (15 F–17 F) that extends into the iliac artery, and a microprocessor-based pump controller, which can provide blood flow up to 4 L/min. The tip of the atrial drainage cannula is positioned under fluoroscopic guidance into the left atrium following transseptal puncture. (b) The Impella LP 2.5 is a catheter-mounted device. The microaxial pump consists of an impeller driven by an integrated microelectric motor on the distal end of a flexible catheter. At a maximum speed of 33,000 rpm, the pump provides a maximum hydraulic capacity of 2.5 L/min. The Impella Recover LP 2.5 is retrogradely placed across the aortic valve into the left ventricle where it aspirates blood via a caged blood flow inlet which is then ejected into the ascending aorta.

One differentiates between long- and short-term as well as surgically implanted versus minimally invasive percutaneous ventricular assist devices (pVAD). The latter have the advantage of availability, simplicity of use, and installation. The present review will concentrate on the two pVADs that have received FDA and CE approval for clinical use, the TandemHeart (Cardiac Assist Inc., Pittsburgh, PA, USA) [3] and the Impella Recover LP 2.5 (AbioMed, Europe, Aachen, Germany) [4] (Figure 1). It also includes a section on the use of extracorporeal life support in cardiogenic shock. 

## 2. Device Specificities, Implantation, and Complications

The *TandemHeart* creates a percutaneous left atrial-to-aortal shunt. Within no more than half an hour, blood is collected from the left atrium, directed to an extracorporeal pump, and then redirected to the abdominal aorta. An operator well trained in transseptal puncture should perform TandemHeart implantation. After gaining femoral venous access, transseptal puncture is performed using standard Brockenbrough technique. When undertaken under cardiopulmonary resuscitation, external cardiac massage should be briefly stopped during a few seconds in order to allow the operator to perform atrial septum puncture. Then, the interatrial septum is dilated using a two-stage (14–21 French) dilator to accommodate the 21-French (Fr) left atrial drainage cannula. Using the Seldinger technique, a 15–17-Fr femoral artery cannula is placed retrogradely in the iliac artery. Both cannulae are connected to the centrifugal pump under careful evacuation of any air within the tubing. The centrifuge is powered by a microprocessor-controlled electromechanical unit, which enables rotation at 3,500 to 7,500 rotations per minute (rpm). The 15-Fr cannula allows a maximal estimated flow of 3.5 L/min and the 17-Fr 4 to 5 L/min depending on systemic vascular resistance. The pump's efficacy also depends on the proper suction of blood from the left atrium, which could be impaired by wedging of the cannula against the atrial wall in case of deep position of the cannula or inappropriate filling of the left atrium. Special care must be taken to avoid displacement or kinking of the inflow cannula, particularly the dislodgement of the cannula from the left into the right atrium. The latter will result in loss of oxygenation and functionally corresponds to a right-to-left shunt. Therefore, the inflow cannula needs to be secured and immobilized in order to minimize the risk of dislodgement. 

Duration of support classically extends from hours to 15 days. When appropriate, a stepwise weaning process (for instance by 500 mL/minute every hour) should be initiated. Weaning criteria are usually met when cardiac index and mean arterial pressure exceed 2.0 L/min/m^2^ and 70 mmHg, respectively, in the absence of end-organ hypoperfusion and without inotropic support. Hemostasis is achieved by manual compression but owing to the large cannula, surgical closure is frequently needed. In comparison, a rather frequent complication (18% of patient with cardiogenic shock at our institution) using the TandemHeart is arterial occlusion and subsequent limb ischemia [5]. Owing to the transseptal puncture, atrial septal defect may persist. Aortic puncture is extremely rare and pericardial tamponade seldom occurs. Finally, particular caution should be made in patients with significant right ventricular failure. Implantation of left-sided TandemHeart might precipitate hemodynamic collapse and death.

The Impella Recover left percutaneous LP 2.5 L/min is a 12-Fr axial flow pump that works on the principle of an Archimedes screw. The impeller is inserted retrogradely through the femoral artery via a 13-Fr peel-away sheath. A 5-Fr Judkins is used to pass through the aortic valve into the left ventricle. The 12 Fr device is then inserted to draw blood out of the left ventricle into the ascending aorta. At maximum speed of 50,000 rpm the pump provides an output of 2.5 L/min. Nine intensities can be adjusted, allowing subtle support. At minimum speed, the pump compensates the aortic regurgitation induced by the catheter. Hemostasis is made by manual compression. The support is weaker than with TandemHeart and usually of shorter duration (from hours up to five days at our institution). However, implantation due to single arterial puncture and familiar technique is faster than TandemHeart. Another is advantage is the absence of transseptal puncture as well as extracorporeal blood flow. Arterial occlusion is infrequent but haemolysis complicated up to 1/5 of patients and typically occurs within the first 48 hours after support begins. 

A similar version, the Impella LP 5.0, achieves a 5 L/min output. The latter requires a surgical procedure [6, 7]. 

**Figure 2:**
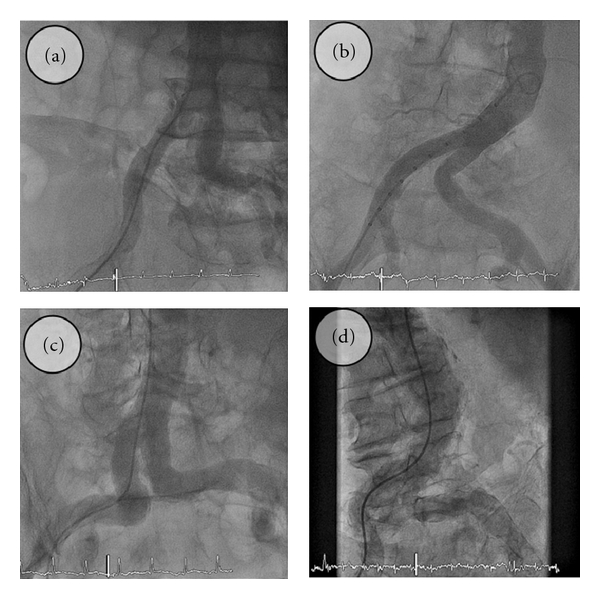
Examples of angiographic assessment prior to percutaneous ventricular assist device implantation. (a)–(d). Suitable anatomy with increasing amount of calcification, plaque, and tortuosity.

Prior to implantation of either device, angiography of the aorta, iliac, and femoral vessels is mandatory in order to evaluate vessel diameter, presence of obstruction, or disproportionate tortuosity (Figure 2). Both pVADs require anticoagulation with heparin at therapeutic levels with recommended activated clotting time of 250 sec during the procedure and 200 sec during support phase. 

Ventricular arrhythmia may complicate the implantation of Impella owing to its intraventricular positioning. A complication common to both pVADs is thrombocytopaenia. Myocardial infarct, atrial cannulation, severe ventricular dysfunction, and postprocedural haemorrhage all contribute to a thromboembolic risk. Infections are usually seen in long-term cardiac assist devices rather than pVADs [8].

Relative contraindications to both pVADs are severe aortic regurgitation, prosthetic aortic valve, as well as aortic aneurysm or dissection. Severe peripheral vascular disease, left ventricular and/or atrial thrombi, severe coagulation disorders, and uncontrolled sepsis further preclude their use.

## 3. Indications

**Figure 3:**
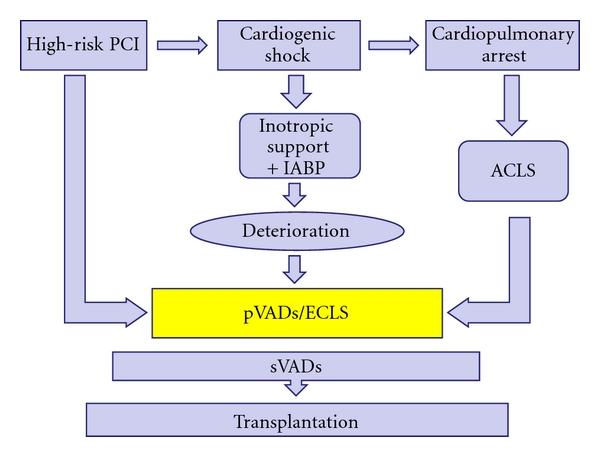
Schematic clinical uses of percutaneous ventricular assist devices (VAD). PCI: percutaneous coronary intervention, ACLS: acute cardiac life support, IABP: intra-aortic balloon pump, pVAD: percutaneous ventricular assist device, sVAD: surgical ventricular assist device, ECLS: extracorporeal life support. Utility of pVADs stretches from prophylactic use in high-risk PCI to immediate life-saving implantation during cardiopulmonary arrest. In cardiogenic shock especially after myocardial infarction, clinical assessment is necessary and current guidelines favour inotropic support with IABP counterpulsation. pVAD or ECLS implantation completes the management when then patient deteriorates. In case of further decline one should think of sVAD as well as heart transplantation.

Cardiogenic shock and high-risk percutaneous coronary intervention (PCI) are two possible indications for percutaneous left ventricular assist devices (Figure 3).

### 3.1. Cardiogenic Shock (CS)

Classically, it is defined on the basis of hemodynamic parameters including systolic systemic blood pressure (sSBP) <90 mmHg for more than 30 min, cardiac index (CI) of <2.2 L/min/m^2^, pulmonary capillary wedge pressure (PCwP) >15 mmHg, and in patients with hypertension a reduction in usual sSBP of >30 mmHg [9]. More importantly it is when cardiac output is severely diminished and responsible for end-organ dysfunction. This enhances neurohumoral responses and systemic inflammatory response syndrome (SIRS) further aggravating the cardiac dysfunction. The incidence of CS in ST-elevated myocardial infarct is unchanged at around 7% [10] and mortality is frighteningly high at 60% despite advances in pharmacological treatment and reperfusion therapy [11]. 

The benefit expected from the implantation of pVADs is alleviation of the strained cardiac muscle and immediate restoration of cardiac output with physiological organ perfusion, thus breaking the vicious cycle of harmful neurohumoral responses and cytokine production. Evaluating the efficiency in terms of evidence-based medicine is problematic considering the small number of patients who benefit from such therapy. However, patient-based, pVAD implantation is undoubtedly life saving and numerous case reports show successful outcomes [12–14]. 

Regarding hemodynamic parameters, evidence shows increased cardiac outputs between 37 to 43% with both pVADs as well as a 38% decrease in PCwP [15, 16]. Clinically, the earlier the assistance is initiated the better the outcome with mortality of 26% when pVADs are initiated in the first 2 weeks as opposed to 40% after 2 weeks [17]. Not surprisingly, outcome is worst in case of biventricular failure with weaning from pVADs decreasing from 73% in left ventricular failure to 53% in biventricular failure [18].

Both TandemHeart and Impella Recover LP 2.5 have been compared to IABP. Two randomised trials have evaluated the TandemHeart in comparison to IABP in patients with CS primarily due to acute myocardial infarction [15, 19]. In both, pVAD improved cardiac index and reduced pulmonary capillary wedge pressure significantly. Importantly there was no difference in mortality and the trials were not designed or powered to assess survival differences. Complications such as limb ischemia and severe haemorrhage were more frequent in the pVAD group than the IABP group. One randomised trial compared the Impella Recover LP 2.5 to IABP [20]. Again, cardiac output was significantly improved in the first group, and there were no differences with respect to 30-day mortality. 

Kar et al. have recently demonstrated the use of TandemHeart in severe refractory cardiogenic shock of both ischemic and nonischemic origin [21]. In this observational study, 117 patients under IABP and/or high-dose vasopressors were implanted with a TandemHeart, 56 of which underwent active cardiopulmonary resuscitation. Mortality rate at 30 days was 40.2% and 45.3% at 6 months. These are significantly lower than the ranges accounted for in previous trials such as the Shock Trial registry. As in previous trials, complications were frequent, amongst which haemorrhage and limb ischemia. 

The Euroshock registry has evaluated the use of Impella Recover 2.5 in 120 patients with cardiogenic shock after acute myocardial infarction [22]. Overall 30-day mortality was 64.2%. The initial hemodynamic profile of patients was poor when compared to other studies reflecting the last-resort use of pVAD. Age over 65 and plasma lactate at admission >3.8 mmol/L were demonstrated to be significant predictors of 30-day mortality. Major cardiac and cerebral events were reported in 15% of patients. 

Although encouraging, these data preclude the use of pVADs as first-line mechanical therapy in cardiogenic shock [23].

### 3.2. Bridging

Significant evidence shows pVAD utility in the bridge-to-recovery concept [24, 25]. This being when the assistance device supports the failing heart in potentially reversible causes of shock such as myocarditis, drug overdose, hypothermia, coronarography-related complications (air embolism, no-reflow phenomenon, and dissections), incessant arrhythmia, or postcardiotomy syndrome. Similarly, pVADs are reliable and used until more definitive measures can be undertaken such as long-term surgical device implantation (*bridge-to-bridge*) and transplantation (*bridge-to-transplantation*) [26, 27].

### 3.3. High-Risk Percutaneous Coronary and Valvular Interventions

Patients with complex coronary artery disease or unprotected left-main coronary artery as well as severe left main coronary stenosis occasionally present with hemodynamic instability or suffer from such comorbidities that they are considered ineligible for coronary artery bypass graft (CABG). Correspondingly, they are at increased risk for hemodynamic collapse during PCI. Preemptive IABP counterpulsation implantation and even cardiopulmonary bypass have been used to anticipate cardiopulmonary collapse management. Recently, investigators have implanted pVADs with the idea of a better supplementation owing to increased cardiac output. Other pathologies necessitating support are critical aortic stenosis and severe cardiomyopathy. 

The first study to have addressed this question showed no significant unloading of the left ventricle [55]. Recently, hemodynamic studies of 11 patients undergoing high-risk PCI with pre-emptive Impella insertion have shown promising results. There was significant left-ventricular unloading as well as decreases in end-diastolic wall stress and improvement in diastolic compliance [65]. 

So far, there is no randomised control trial, but many observational, retrospective studies show safety of use, little device complications, and lower than predicted mortality at 30 days [56, 66, 67].  Table 1 summarizes the in-hospital survival of patients having undergone high-risk PCI with pVAD implantation. It also shows in-hospital survival of patients with cardiogenic shock due to acute myocardial infarction treated with either surgical or percutaneous ventricular assist devices.

The Europella Registry published a retrospective study with 144 patients. Thirty-day mortality was 5.5%. 6.2% of patients had bleeding and 4% vascular complications [68]. Recently, a randomised controlled study, Protect II, compared the use of IABP to Impella Recover 2.5 in high-risk PCI in 305 patients. Abiomed stopped the trial at the end of 2010 after determining it could not reach its composite primary end-point of 10 major adverse events. Provisional results failed to demonstrate the superiority of the Impella Recover 2.5 LP [69]. 

Therefore, the prophylactic use of pVADs in high-risk PCI and other interventions, however appealing, should be considered with caution until further evidence is published.

### 3.4. Ventricular Tachycardia Ablation

VT ablation is increasingly performed particularly in patients with structural heart disease, for symptom management or in the case of frequent ICD shocks. In the hemodynamically unstable patient, substrate-based approaches allow successful ablation without inducing arrhythmia. However, when this approach fails it may be difficult if not impossible to ablate hemodynamically unstable arrhythmias. A number of case reports demonstrate the benefit of pVADs to achieve hemodynamic stability and allow successful procedures. TandemHeart was first used in 2007 as a support for VT ablation in a 55-year-old man [70]. Later, unstable VT ablation was successfully achieved in 3 patients using Impella Recover 2.5 LP support [71]. Further case reports have been published including the use of pVADs in other types of arrhythmias such as unstable supra-ventricular tachycardias in the setting of congenital heart disease [72, 73].

## 4. Right Ventricular and Biventricular Assistance

Acute right ventricular (RV) myocardial infarction may result in ventricular wall dysfunction and dramatic effects on biventricular performance. Transpulmonary cardiac output is diminished thereby compromising left ventricular (LV) preload resulting in overall diminished cardiac output. The RV dilates and pericardial pressure increases, changing LV compliance via ventricular interdependence. Classically, management other than rapid reperfusion consists of volume resuscitation and inotropic support. Little is known on the use of pVADs in RV failure and, as noted above, left-sided pVAD such as TandemHeart are contraindicated in this setting as they aggravate the fragile hemodynamic equilibrium. However, dedicated TandemHeart cannulae have been developed for the right ventricle (pRVAD). One initial case report demonstrated the feasibility of pRVAD with Tandem Heart [74]. Another case report shows successful 3-day support with an adapted TandemHeart (pRVAD) [75]. In both cases, the chosen cardiac output was a maximum of 3.5 L/min with mean between 2 and 3 L/min. Successful bilateral percutaneous assist device support was accomplished via pRVAD with TandemHeart and left IABP counterpulsation in an acute biventricular myocardial infarction. The patient was under mechanical support for 48 hours and was discharged eight days after the procedure [76]. Finally, biventricular support with pRVAD TandemHeart and pLVAD with Impella Recover LP 2.5 allowed complete recovery of a patient with severe cardiac allograft rejection [14]. Admittedly, these are isolated cases in which last resort complex and potentially dangerous procedures were initiated. They nevertheless emphasise the life-saving potential of pVADs.

## 5. Extracorporeal Life Support

**Figure 4:**
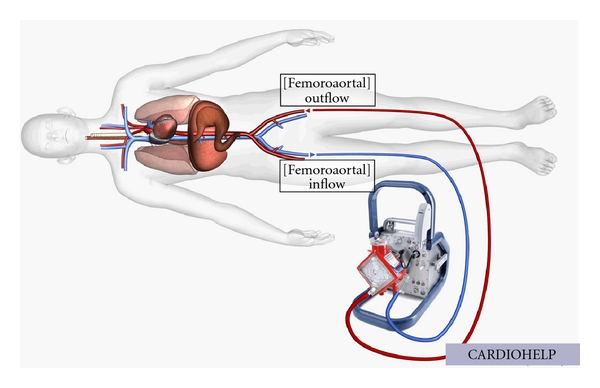
Example of extracorporeal life support (ECLS). CARDIOHELP System (MAQUET, Cardiopulmonary AG, Germany). Minimised hand-held ECLS with representation of a femorofemoral, venoarterial cannulation. Deoxygenated blood is harvested in the femoral vein and pumped back to the iliac artery after having passed through the oxygenating membrane and the heat exchanger.

Extracorporeal life support encompasses life support devices including oxygenation, carbon dioxide removal, and hemodynamic support. It is a form of cardiopulmonary bypass allowing either lung, or both lung and heart support. The basic circuit consists of a venous cannula harvesting deoxygenated blood, a 4000 rpm centrifugal pump with up to 7 L/min high flow, a membrane oxygenator, a heat exchanger, and a returning cannula with oxygenated blood. Two distinct configurations exist, one being a venovenous (VV) cannulation bypassing the lungs and allowing support in respiratory failure. The other being the venoarterial (VA) cannulation where the oxygenated blood is pumped back to the arterial system bypassing lungs and heart providing not only respiratory but also hemodynamic support (see Figure 4). Only the veno-arterial cannulation within the spectrum of hemodynamic support will be considered here. 

Technically, the extracting, 22–30-Fr venous cannula is inserted using the Seldinger technique in the right common femoral vein. The 15–23-Fr arterial cannula is placed in the right common femoral artery and maintained in the iliac artery. A supplementary arterial cannula may be inserted distal to the femoral artery cannula to prevent lower limb ischemia. If the lower limb vessels are unsuitable, right common carotid artery or axillary artery cannulation is possible. 

Anticoagulation is achieved through continuous unfractionated heparin infusion with recommended ACT between 210 and 230 seconds. Platelet count should be maintained greater than 100,000/microL as sheer forces and exposure to foreign body continuously consume them. The duration of support is classically described from 15 to 21 days for femoral access and up to two months for central thoracic access. Complications include local hemorrhage, thromboembolism, lower limb ischaemia, ischemic and hemorrhagic stroke, haemolysis, and infections. 

Special attention must be made when cardiac function recovers with flow competing against the ECLS returning blood in the aorta. In case of persistent respiratory failure, the Harlequin syndrome classically describes a blue-headed (deoxygenated blood directed to the upper body) and red-legged patient (hyperoxygenated blood to the lower body). Switch from VA to VV ECLS may then be needed. 

Indications range from severe refractory cardiogenic shock [77], cardiac arrest [78] to failure to wean from cardiopulmonary bypass in cardiac surgery [79] and finally as a bridge [80] to either transplantation or sVAD. Relative contraindications are similar to those for VAD as stated above. 

To date, there have been no randomised trials assessing ECLS efficacy in hemodynamic support but observational studies exhibit promising results. 

Two studies showed a benefit of ECLS performed in cardiac arrest [81, 82]. Short-term and 6-month survival rate were significantly increased in 59 and 85 patients under ECLS-CPR as compared with conventional CPR. Another study evaluated the outcomes of 81 patients who benefited from ECLS in severe refractory cardiogenic shock with long-term survival rates of 36% [77]. In comparison to biventricular assist devices, ECLS was as effective in recovery of fulminant myocarditis yet with faster renal and hepatic recovery [83].

Newer, minimised ECLS systems such as the ELS-System and Cardiohelp (both from MAQUET Cardiopulmonary AG, Germany) have been developed allowing rapid insertion and facilitated interhospital transport [84]. One case report showed safe application of Cardiohelp in 6 patients. Interhospital transport was done by car or helicopter and survival rate was 100% [85].

## 6. Future Devices

Another promising device not commercially available is the Reitan Catheter Pump (RCP; Kiwimed Ltd.). It consists of a catheter-mounted pump-head with a foldable propeller and surrounding cage. Positioned in the descending aorta, the pump creates a pressure gradient, reducing afterload and enhancing organ perfusion. One study confirmed its safety in 11 high-risk PCI patients [86]. Benefits on hemodynamic parameters especially cardiac output have not been shown in humans.

## 7. Conclusion

Acute heart failure and cardiogenic shock, regardless the cause, still have a dreadful outcome. Current management includes use of inotropic support and/or IABP. In the past decade, pVAD and ECLS have completed this armamentarium with which one can tackle these conditions. A true technological advance, proven to restore and maintain perfusion pressures. Although better hemodynamic parameters are interesting, improved clinical outcome has yet to be demonstrated. Ultimately, complications arising from insertion and costs further broaden the debate. In this sense, pVADs and ECLS are no plot devices, and the seemingly inextricable problem of acute heart failure is not likely to be solved with a sole object.

## Figures and Tables

**Table 1:** Early clinical outcome in (A) patients with cardiogenic shock and treated with surgical or percutaneous ventricular assist device (s- or pVAD) and (B) in patients after preventive pVAD implantation for high-risk percutaneous coronary intervention (PCI).

	Device	*N* of patients	30-day survival
A. (Cardiogenic shock)	sVAD^a^	157	92 (59%)
	pVAD^b^	102	65 (64%)
B. (High-risk PCI)	pVAD TH^c^	113	94 (83%)
	pVAD IP^d^	152	59 (78%)

TH means TandemHeart, IP is for Impella Recover 2.5 LP; (a) pooled data from 11 trials [17, 28–37], (b) pooled data from 11 trials [5–7, 15, 19, 38–43], (c) pooled data from 11 trials [44–54], and (d) pooled data from 10 trials [55–64].
